# Collectanea Medica, Consisting of Anecdotes, Facts, Extracts, Illustrations, Queries, Suggestions, &c.

**Published:** 1816-08

**Authors:** 


### COLLECTANEA MEDICA,

CONSISTING OF

### ANECDOTES, FACTS, EXTRACTS, ILLUSTRATIONS, QUERIES, SUGGESTIONS, &c.

RELATING TO THE

*History or the Art of Medicine, and the Auxiliary Sciences.*

> Quicquid agunt medici,

> Nostri farrago libelli.

DIABETES, with other diseases heretofore considered incurable, has, within these few years, much attracted the attention of the medical world. Can there be a stronger proof of the advancement of our art? Even the very recent communication from Mr. Smerdon, in our last Number, has already become a subject of conversation in London. One gentleman, who has the care of two very extensive public charities, and whose diligence, fidelity, and experimental knowledge is beyond all scruple, assures us that he has seen three cases very lately cured by tonics, some of which had become so much worse by bleeding, that the patients refused to submit any longer to that practice. All our readers must recollect Dr. Warren's success with large doses of opium, contained in the 4th vol. of Medical Transactions. We now transcribe the following from the last volume published by the same learned body, that our readers may have every opportunity of solving the following problems:

1st. Does diabetes arise from various causes, and consequently require different modes of treatment?

2d. Is the opinion of the late Dr. Heberden well confirmed, that diabetes is for the most part only symptomatic of some morbid irritation, distinct from the kidneys; and is it sometimes only one of that complicated train of symptoms which ends in dissolution?

3d. Are there always diagnostic symptoms independent of the

 urine, urine, and should we not look for the cause and seat of the disease in those other symptoms?

*Cases of Diabetes;* by Richard Patrick Satterley, M. D. Fellow of the Royal College of Physicians, and Physician to the Middlesex and Foundling Hospitals.

The paper which has been recently read before the College, on the subject of Diabetes, by my friend Dr. Warren, suggested to me the propriety of submitting to their consideration the following cases, in which a very opposite mode of treatment has been pursued. As these are brought forward rather on account of the peculiar practice employed, than in illustration of the nature of the complaint itself, I have confined myself to that detail of symptoms which might be sufficient to prove the identity of the disease, and the effect of the remedy.

In these cases, especially in the first, or principal one, if any doubt could be entertained, either of the nature and extent of the disease, or of the value of the remedy, I might appeal to my friends and colleagues, Dr. Gower and Dr. Price, under whose observation the whole cure was conducted, and without whose cordial sanction and assistance, I might have hesitated to have resorted to such vigorous measures.

John Manley, 32 years of age, was brought to the Middlesex Hospital on the 19th of February, 1808. He appeared wan and dejected, his countenance was sallow, and his strength very much diminished; he complained of great thirst, a constant desire for food, and an uneasy sensation at the pit of the stomach; his eyes were peculiarly white, his tongue was thickly covered with a milky fur and was very dry, his gums were of a dark colour and spongy, and his teeth were loosened; his skin was harsh and dry, and so lax, that it might be gathered up in folds; his body was costive, and he suffered much from a prolapsus of the anus. He passed his urine frequently, being obliged to get out of bed ten or twelve times in a night. He complained of chills, succeeded by burning heats; and his pulse was quick, very small, and hard. The most urgent symptom was a pain in the loins, which he described as always severe, but at times excessively acute. His testicles also were occasionally retracted. For more than three months he had lived in the poor-house, unable to make the slightest exertion; indeed his debility was so extreme, that without assistance he was unable to walk to the ward. He had been ill nearly six months, and ascribed his complaints to his having drunk copiously of cold water when over-heated, a circumstance which gives great support to the opinion of Dr. Mead, who attributes the greater prevalence of diabetes among the moderns than among the ancients, to this very cause of quenching the thirst by cold instead of warm liquids.

I merely directed that ℥ifs. of castor oil should be taken immediately, and repeated if necessary. On the following morning I found that in the preceding 24 hours, he had passed 16 quarts of urine, which had the violet smell, was sweet to the taste, and which yielded, on evaporation, from each pound, more than ℥iſs. of a black treacle. When the urine was first made, very minute globules of an unctuous substance were seen floating in it, which, as it cooled, collected in large quantities on the surface. This, no doubt, was the oil, which is mentioned by authors as observable in diabetic urine.

Considering that whatever might be the cause of the increased flow of urine, there was strong evidence of some inflammatory action in the kidneys; and finding, from the able work of Dr. Watt, of Glasgow, that even in diabetes itself, the lancet had been usefully employed; I might further add, considering the hopeless state of my patient, I did not hesitate to determine on a trial of the effect of blood-letting: ℥xiv. of blood were accordingly drawn from the arm, and a large blister was applied to the loins. He was ordered a meat diet, and was directed to limit his drink to that quantity which might be adequate to allay, though not to satiate, his distressing thirst. In the 24 hours subsequent to the bleeding, he had passed 11 quarts of urine only, and had restricted his drink to eight, three pints of which had been lime-water. The pain in the kidneys was very little abated, and his pulse was hard, but not increased in frequency; he expressed a sense of relief, and was fully satisfied that he was better and stronger. The appearance of the blood was peculiar; it was a homogeneous mass, in colour almost black, and in it no separation of serum and crassamentum was distinguishable. It had so little cohesion, that a fork passed through it with no greater resistance than through treacle, to which indeed it bore a much nearer resemblance than to blood. He was again bled to ℥xviij, and a grain of calomel, with a few grains of the compound ipecacuanha powder, were given every night; and the castor oil was repeated as often as it appeared to be necessary. I saw him on the following morning: he had borne the second loss of blood as well as the first; he neither felt faint or weaker, but expressed the same sense of relief as before. The pain in the kidneys was lessened, and the urine was reduced to six quarts. The blood looked more natural in its texture, and a slight separation had taken place after it had stood a few hours.

On the 23d, the quantity of urine was increased to nine quarts, and the patient seemed heavy and dull: ℥xx of blood were taken away, and the other remedies continued; the urine was reduced to six quarts on the following day, but on the 25th the patient was more irritable, complained that his thirst was immoderate, that his night had been restless, that his skin was more dry, and that the pain in the kidneys was more acute; ℥xx of blood were taken, and as the castor oil failed in keeping the bowels lax, although he took six and eight ounces a day, some aloetic pills and the common purging draughts were substituted for it, and the compound ipecacuanha powder was directed to be taken every six hours. The blood was of much firmer consistence, and more natural tural in its appearance; the crassamentum was covered with a membrane analogous to the buffy coat, but of an intensely bright scarlet colour. He experienced great comfort from this loss of blood; and on the 28th I learned that the urine passed in 24 hours had varied for several days, from five to seven quarts, at which last quantity it was on that morning. He seemed more uneasy and complaining, but he was able to walk about the ward, and was fully confident of recovery: ℥xviij of blood were drawn, and, as he was tired of lime-water, alum whey was substituted. I saw him again on the following day, when he expressed great satisfaction at the relief he had experienced from the last bleeding. The pain in the loins he described as greatly diminished, but not altogether removed; his thirst was less urgent, and his night had been comfortable; for the first time he observed a little moisture on the skin, his tongue had gradually become cleaner, but it was still white. On the 3d of March, his urine measured six quarts; on the 4th, seven and a half: his countenance was more cheerful, he was in excellent spirits, and his strength so much increased that he walked out into the garden; but, as his pulse was more frequent and hard, and as the pain in the loins was rather increased, ℥xviij of blood were again taken away, which presented the usual appearances of inflammatory blood. From this day till the 11th, the urine passed varied but little, never exceeding six, or falling short of five, quarts; but his bodily strength daily increased, his thirst abated, and his nights passed without any inclination to make water; but, as he still complained of pain in the loins, and his pulse was hard, ℥xviij of blood were drawn on that day. By this last bleeding he was more benefitted than by any of the former,—the sense of weight and pain in the loins was removed, and the urine reduced below five quarts. The blood was still very much buffed. The immediate relief he obtained from each bleeding was so great, that he begged more frequently for a renewal of the remedy than I deemed it prudent to grant, and would often compel the apothecary to take away several ounces more than the prescribed quantity.

His appetite, which at first was ravenous, became more moderate, and his thirst was so reduced, that the ordinary quantity of drink served to satisfy it, and the urine was gradually reduced from five to three and two quarts, and all the other symptoms vanished. Several evaporations of the urine were made during the progress of the cure, and the quantity of saccharine extract was found to diminish in proportion as the urine itself was diminished; so that a much less quantity of extract was discoverable in each quart when the urine measured six quarts, than when it was high as 12 or 16; but, even when the urine was reduced to two quarts, sugar was found in it. He continued in tolerably good health, and increased in strength and size, until the end of the month, when, without any particular exposure, pain in the chest and other slight symptoms of pneumonia came on, which readily yielded to one or two two additional bleedings. He continued a very long time in the hospital, but was ultimately discharged cured, and I have been able to ascertain that for *two years* he remained in good health (and I have reason to believe up to the present time), and that he has *never* experienced a recurrence of the disease.

The next patient under my care was a poor woman in the country, 30 years of age, who had been ill for three months. She complained of frequent and irregular cold shiverings and transient but burning heats, which were now and then followed by perspiration. Her appetite was immoderate, for scanty as her means were, she was obliged, from a constant craving, to eat every hour. She was dreadfully emaciated, and her bodily strength so far reduced, that she could with difficulty walk a hundred yards. Her pulse was 110 and hard, her thirst insatiate, her tongue dry and highly reddened, except at the root, which was covered with a thick milky fur; her throat was dry and sore, and she complained of a distressing itching of the pudendum; the inclination to make water was frequent, and she estimated the quantity passed in 24 hours at seven quarts, but, for the two preceding days, having limited her drink to three quarts, her urine had not exceeded five quarts; it was of a violet smell, and straw-coloured, and yielded largely of saccharine extract. She also complained of pain in the small of the back, and was irregular in menstruation. The success which had followed the use of the lancet, in the preceding case, determined me as to the plan I would adopt, and ℥xij of blood were drawn, the bowels were kept open by castor oil, and the carbonate of ammonia, with a slight opiate, were given two or three times a day. During the bleeding, the patient fainted, but after her recovery she did not seem weaker than before the operation. The blood, when it first flowed, was of the same character as that of Manley, but after a few hours a separation took place as in health; the proportion of crassamentum was greater than usual, but the substance and texture of it were less firm. The flow of urine was somewhat diminished by the bleeding, and the sufferings of the patient much lessened. In two days afterwards she was bled again to about ℥xvj, and from this second loss of blood, the urine was reduced to three quarts. She gradually lost the diabetic symptoms, and continued free from the complaint until her death, which took place after a lapse of some months.

The cause of her death, which happened during my absence from Tunbridge Wells, I am unacquainted with, but I have ascertained that it was unattended by any increased flow of urine.

The marked good effects of bleeding in diabetes was further confirmed in the case of a patient of rank, under the care of Sir W. Farquhar, Mr. Kelson of Sevenoaks, and myself. A constitution previously diseased and weakened by a residence in hot climates, had rendered this lady as unfit a subject for the lancet as could well be conceived. She had returned from India five or six years, labouring under the usual indispositions resulting from vitiated vitiated and irregular biliary secretions, and continued suffering a variety of ailments, in the autumn of 1810, until they imperceptibly assumed the form of diabetes. Before I saw her, she had for many weeks suffered from intense thirst, great dryness of the skin, and a coated and foul tongue, which was excessively parched and rough in the morning; her pulse was hard and rapid, but contracted; she was greatly emaciated, her anxiety and restlessness were very remarkable, her inclination to make water was incessant, and the quantity passed considerable; it was of a violet smell, and yielded the treacly extract: she had also the same uneasiness and pain in the loins as Manley: ℥xij of blood were drawn, which diminished the quantity of urine one-half, and produced a considerable alleviation of every symptom, and three repetitions of this remedy removed all traces of the complaint.

I have yet one other instance to adduce, in which the lancet was serviceable, though not to the same extent as in the former cases.

A woman, 40 years of age, but so emaciated and enfeebled that I guessed her to be 70, had for years laboured under diabetes, and generally passed from 10 to 14 quarts of sweet water daily. She had the usual symptoms of parched skin, rough and hard tongue, rapid pulse, and distressing itching of the pudendum; the pain in the loins was considerable: ℥xiv of blood were taken, and a syncope of half an hour's continuance resulted. The blood was precisely in the same state as that of Manley, as dark as treacle, and showing so little disposition to coagulate, that after it had been drawn near an hour, it would flow from the bason like water. This patient's miserable abode was at a great distance from me, and I had not an opportunity of seeing her a second time; but a very respectable apothecary, Mr. Stone, of Mayfield, reported to me that the water was reduced full one half. The wretchedness of this poor creature precluded all possibility of her compliance with any regular diet or regimen, and she could not be persuaded to consent to a repetition of the bleeding, although such beneficial effects had followed the first operation.

This is the extent of my experience respecting bleeding in diabetes; an experience that fully warrants my asserting the safety, and, I think, the efficacy, of the practice, in some species of this complaint. The success which has attended two such different modes of treatment as the one pursued by my friend Dr. Warren, and that which I have related, might afford matter for much reasoning. It shows that nature is capable of attaining, by various means, the same end; or that the copious discharge of urine, like the accumulation of water in ascites, must often be a symptom arising from very opposite affections, and that the inflammatory diathesis is more frequently present in the constitution than is commonly suspected.

On

*On the Properties of Life;* by JAMES JACKSON, M.D. Professor of the Theory and Practice of Physic in Harvard University.

SOME of the opinions advanced in the following paper may have claims to novelty; but for the most part they are destitute of such claims, and will be found to correspond with the doctrines of the best physiologists of the present age. They are offered to the medical public as containing simple views of a subject, on which all must have reflected, but on which perhaps few members of the profession have established in their own minds clear and distinct opinions. In stating this, it is not intended to intimate that the reader will find himself fully satisfied by the exposition, which will now be given, of a subject shrouded in much darkness. The human mind is never content to rest long in the pursuit of causes; and at whatever point it may arrive, it will experience an eagerness to explore still further the unknown regions before it. The attempt in this paper will be to give a definite statement of the knowledge already attained in respect to the subject of vitality. Future experiments and a more accurate analysis may reduce to a smaller number the elementary principles of this department of knowledge. True philosophy teaches us not to multiply causes; but it inculcates also a precept which is still more important. This is that, in the analysis of phenomena, we should be careful not to refer them to principles, which are too few and too limited to explain the occurrence of those phenomena. An accurate analysis, the true inductive philosophy can never lead to such an error. Common language shows that most physiologists have attributed the phenomena of life to a single power, which has been denominated the *anima, the principle of vitality*, &c. Physiologists have often rested on this opinion as on a basis generally admitted, and have troubled themselves only to ascertain in what organ, or in what subordinate system of the body this principle has its residence; and in what manner other parts of the body derive their powers from it. Now it will not be attempted to disprove this opinion respecting the unity of the vital power. It may be allowed us to pass by this popular hypothesis until some evidence is adduced in support of it, and meanwhile to enquire what can be proved in respect to the powers or properties peculiar to living bodies. These powers may be denominated properties of life, or vital properties. In enumerating those, the existence of which can be proved, we do not deny the existence of others. Nor, in so doing, do we deny that a further analysis may show that two or more of these these powers may be referred to some one of a character more general.

Whatever can be known in respect to the living body, may be comprehended under the heads of its composition, its structure, its functions, its appetites, and its vital properties.

In its composition we regard the ultimate elements, into which it may be decomposed when deprived of life, and the various combinations of these elements, which exist in it during life.

In its structure, we regard the texture of the different parts of the body, and the combination of parts differing from each other in texture, together with the form or configuration produced by such combinations in the various organs. In respect to the form of the organs we find it possible to make imitations with common matter; but not so in respect to their texture. We only approximate to a knowledge of their textures, and from this cause it is impossible to imitate them. But there is another reason why we cannot imitate them; and that is, because we cannot procure materials of the same composition.

It is not necessary to our present purpose to make any further remarks in respect either to the composition, or to the structure of the living body; for these are both produced by the functions. It is by the functions of the parent that the offspring is first produced, deriving from that source a certain composition and a certain structure. Afterwards by its own functions it converts foreign materials to its own purposes, giving to them the requisite composition and structure. To the performance of some of the functions we are prompted by the appetites; which may perhaps be considered as properties of vitality. We shall not however argue this matter at present. In the performance of some of the functions much is to be attributed to the configuration and relative position of the various organs; and hence arise many mechanical relations between different parts of living bodies. Hence the configuration and relative position of different organs are to be regarded as of the utmost consequence in explaining the functions, and in explaining the disorders which may take place in them. But neither need these subjects be discussed for our present purpose.

In order to the performance of the functions it is not enough that the organs maintain certain forms and be composed of parts of certain textures, and that they contain matters proper to be acted upon. All this may be the case in a dead body. To the performance of the functions it is requisite that, in addition to the conditions above described, the the organs possess certain powers peculiar to living bodies. Now, what these powers are, we must learn from an examination of the functions. Let us then first enumerate the functions performed in the human system, and then enquire what powers are demonstrated in the various organs by the performance of these functions. In making this enumeration we shall follow Bichat in his great divisions, but not altogether in those which are subordinate.

Bichat divides the functions into two classes; the first containing those which appertain to the individual; the second those which appertain to the species.

The functions of the first class he divides into two orders, the organic and the animal.

I. The organic, sometimes called the internal functions, are common to all organised beings. They relate to the individual himself, and may be arranged under three genera, viz.; those of assimilation; those of formation; and those of excretion. In these functions generally we find hollow organs acting upon their contents; which contents, except in the alimentary canal, are in a fluid state.

In the functions of assimilation foreign matter is converted into a fluid, which is distributed to every part of the living body, distending its vessels and furnishing materials for the supply of its waste and for the renewal of its secretions. These functions are effected by very different apparatus, and are accordingly more or less complicated in different living beings. In animals, a stomach appears to be essential for the purpose, but this purpose is completed within the circulating vessels. In plants, it is effected by the vessels alone.

The functions of formation are performed by the extreme vessels. In health the capillaries always hold in readiness the blood, from which the vessels which perform exhalation, secretion, and nutrition, immediately derive their supplies. This is true at least of all large animals.

The excretory functions are performed in part by processes analogous to secretion. This is true however only of those excretions, which take place from the blood. Those excretions in animals which take place from the alimentary canal, consisting principally of matters which have never properly entered into the living system, are performed by different organs. On this head it is not requisite to be more particular.

II. The animal functions are such as are performed only in animals. They are not all of them however performed by all animals. They have been called the external functions; yet certainly some of them, viz. some of the intellectual functions, functions, cannot be said to relate to external things. This remark will not probably be controverted in respect to the exercise of consciousness, which is in every view an internal function. In the performance of the animal functions we have not any evidence that there are hollow organs, which act upon their contents; nor indeed that there take place any mechanical operations in the organs, except in locomotion and in the voice. The animal functions may be divided into three genera, viz. those of sensation; those of the mind, or intellectual functions; and those of motion under the control of the mind.

It is not necessary to remark here on the characters of these functions.

The functions of the second class are divided by Bichat into three orders. Under the first and second are included those of the two sexes; under the third that common to both. In these functions we find some, which are analogous to those called animal, and others analogous to those called organic.

These several functions are not independent of each other. Various relations are maintained between them. These relations can, some of them, be explained upon principles which are called mechanical; others can be explained only upon principles peculiar to living bodies, and may therefore be denominated vital relations. The vital relations are some of them maintained through the medium of certain organs, viz. the nerves, interposed between those organs by which the functions so related to each other are performed. These relations may be called nervous. Other vital relations are performed between parts, which have not any peculiar connection with each other. It is probable that they are maintained through the medium of the encephalon, spinal marrow, and nerves. By these relations we find one part suffers either pain or pleasure from an affection of another part; or one part has its actions arrested, diminished, increased, or modified, in consequence of the affection of another part. From the analogy manifested in these relations between different parts to those noticed between different individual animals, these relations have been called sympathetic.

These remarks on the functions are sufficient to enable us to explain the vital properties, which are exercised in the performance of them. In describing these properties it is not requisite to pursue the rigid analytical process, by which their existence may be discovered. It will be enough to describe the different vital properties, and to show that these are exercised in the various functions.

The vital properties, whose existence is most clearly ascertained, tained, are the following, viz. mobility, irritability, vital affinity, vivification, sensibility, sympathy, and the intellectual powers.

Mobility is the power of original motion in living beings. It resides most especially in the muscles; but it is not confined to them, as it is found to take place in parts destitute of the peculiar structure of muscles. It cannot be necessary to show that the motions performed by our muscles do not arise from impulse; that these motions are not communicated to the muscles from any other bodies in motion. Neither is there any reason to attribute the motions in living bodies to any of those powers called attractions, which occasion motions in inanimate matter under certain circumstances; such as the attraction of cohesion, of electricity, and of galvanism. Whether mobility is a property inherent in the muscles, &c. or is derived to them from other organs, are questions not requisite to be decided for our present purpose. It is enough to show that it is a property belonging to living bodies. It is a property manifested both in the organs, which perform the organic, and in those which perform the animal functions; and likewise in those performing the functions of the third class. The motions which are performed in these various organs, are some of them very sensible; others are insensible. The performance of these last is manifested by their effects. These motions are insensible, because the organs performing them are extremely small, and the extent of their motions too limited to be measured by our organs of sense. The presumption is, that the power of motion, or mobility, is the same in all the organs enjoying this power, unless some evidence of a difference in different organs can be demonstrated. Mr. Bichat has however distinguished this property in different organs, by different names, without any such demonstration.

Irritability is the property of being excited to motion by the influence of something exterior to the moving organ. This is a property closely allied to mobility, and it is spoken of by many physiologists as comprising this latter property. The two properties are however evidently distinct in their natures. Irritability is indeed always found to exist in the organs possessing mobility. Without the former, the existence of the latter could never be discovered; for parts possessed of mobility never perform spontaneous motion, but are always excited to motion by the agency of external causes called stimuli. Now stimuli act on the irritability. But it is not necessary that stimuli be applied to the organ excited to motion. From this we find that irritability may reside in parts which do not possess mobility; and from this circumstance, circumstance, were it not otherwise shown, we might infer that the two properties are distinct. When a stimulus occasions motion in the intestinal canal in a sound state of the parts, it is applied to the mucous membrane or coat, and not to the muscular coat, by which the motion it performed. There is a common case which is analogous to this, but in which the parts are much farther removed from each other. This is the sudden and violent excitement of the respiratory muscles in sneezing, in consequence of the application of a stimulus to the mucous membrane of the nose.

Irritability is variously modified in different organs; being adapted in each to the appropriate stimuli of each. The various parts performing the organic functions have each their respective stimuli. And although the same stimulus will act on two or more of those parts, it will produce in them different effects.

It is supposed that this same property, irritability, is acted upon, when the functions of the secretory organs are induced by any cause, or agent, influencing those organs. This supposition is connected with the opinion that secretions are, in part at least, performed by certain motions of the small vessels. Highly probable as this opinion is, it is rather an inference than a fact demonstrated.

The next vital property, which was enumerated, is one which has not been distinctly recognised by physiologists; although the facts, from which the existence of this property is inferred, have been duly regarded in modern days. To the property in question I have ventured to give the name of vital affinity. In respect to inanimate matter it is well known that different bodies, whether elementary or compound, exercise mutual attractions, or affinities; and although the force of these affinities differs materially in different cases, yet the power is thought to be of the same kind in all cases. The principal objects of chemistry are to ascertain these powers and to measure their relative strength. The power exercised in every such case is called chemical affinity. Now the ultimate elementary particles in living bodies are the same as those in common matter, or inanimate substances. These particles in living bodies are combined by affinity or attraction; but they are not combined in the same manner as they are in inanimate matter. When they become parts of a living body they are controled by new affinities. This power is of one kind in all these cases, and being analogous to chemical affinity, yet differing from it, there does not seem any more appropriate name for it than the one above-mentioned, viz. vital affinity. The evidences of the difference in the combinations in living and common matter, ter, are so numerous and so familiar, that it cannot be necessary to detail them. It must be sufficient to refer to the decomposition, which follows death in both animal and vegetable bodies. This decomposition results from the new arrangement of the elements of those bodies, when those elements are restored to the influence of chemical affinity. Had they been combined by chemical affinity in the living body they would not undergo decomposition; for the dead and living bodies are placed in the same situation, or exposure, as regards the action of external causes. It is probable that vital affinity is first communicated to foreign matter in the process of digestion; that, when the chyle enters the blood-vessels, some part is thrown off, or separated from it by some of the emunctories; and that the parts which are left are thereby so varied as to form blood. It may be, however, that the affinities of the elements of chyle are changed in some measure in the blood-vessels, and that thus the same elements are made to form a new compound. Be this as it may, it is certain that when the nutriment has become perfectly assimilated, so as to form blood, the elements which had first constituted that nutriment and now constitute the blood, have had their affinities changed. It is true that the solid articles and some of the liquid articles, which are employed for the nutrition of man and of other animals, consist of matter which has recently enjoyed life, and of which the elements have not yet become arranged, or combined in accordance with their chemical affinities. No doubt the assimilation is thus facilitated, and that the assimilating powers of man and of most other animals are not sufficient to enable them to change the affinities of the elements in common matter. Man for instance cannot get nourishment from air and water, although these contain the principal ingredients which compose his body. But there are a vast many living beings, viz. plants, which can assimilate to themselves such common matter, and it is from such originally that those substances have been formed which serve for nutriment to man. And in respect to those substances which serve us for nutriment, though their elements are combined by the general power of vital affinity, yet their combination is very different from that of the same elements in chyle and in blood. Their conversion is effected by the assimilating organs, whence those elements receive new vital affinities.

Very similar changes take place in the formative functions. By these functions the various solids and various secreted fluids of our bodies are formed. They are formed from that heterogeneous fluid called the blood. In one instance, that of fibrine, or muscular fibre, the matter formed seems to consist consist almost precisely of coagulating lymph changed from its fluid to its solid state. This is one of the primary elements of the blood. But in respect to other parts formed from the blood, nothing like this appears to be true. In respect to all such parts then it would seem, that the matter which composes them, has its elements endued with new vital affinities in the acts of secretion and of nutrition. Vital affinity is not in these functions given *de novo;* it is only changed or modified. This modification can be effected only by the extreme vessels in the respective parts; for to those vessels only are those substances exposed, which undergo the change.

The fourth property is another to which I have ventured to supply a name. The conversion of matter from the inanimate to the animate state is among the most wonderful and unaccountable operations in nature. When a piece of iron is rendered magnetical by the application of a magnet, or when one body is electrified by another previously in that state, we can conceive that these changes are wrought by the transmission of some matter from one body to the other, or by a new arrangement in the constituent particles in the body which undergoes the change. But when matter, which was inanimate, is received into a living body, becomes a part of it, and acquires its peculiar powers, we cannot carry our explanations beyond the distinct statement of the facts. That which lives has the power of making other matter live. It has the property of vivification. Its various parts have this property so modified, as to give to new matter the various properties of those parts respectively. While the matter of living bodies is continually changing, and individual beings are each in turn losing their vitality, as regards their material systems, these vital powers are preserved in perpetual existence. They were given to the first created beings, and they have been transmitted from them through the successive races of their offspring, and seem capable of being extended and renewed indefinitely. In the formation of every new part, the organs of formation, while bestowing on such part its structure and its composition, also bestow on it its appropriate vital properties. In so doing, those organs exercise a vital property, and it is to this I have given the name of vivification.

The four properties which have now been described may be found in every living organized being. These properties are perhaps all that are necessary for the performance of the organic functions. The third of these properties, vital affinity, must exist in every part of matter which enjoys life. It is the last property which is lost after general death takes place, place. The other three properties are obviously confined to particular organs. Mobility exists in the muscles, in the vascular organs, and in all other parts which perform motion. Irritability exists in the same parts, and likewise in the various surfaces of the several organs which are exposed to the action of stimuli in the common functions. Vivification exists in the minute vessels which perform the formative functions, or the functions of nutrition and secretion.

But all these parts, and even the fluids themselves, are possessed of vital affinity. Were not the elements of our fluids, as well as of our solids, combined by affinities not chemical, the former would not, like the latter, be subject to decomposition after death.

Sensibility is the fifth property which was mentioned. We have not any reason to believe that this exists in any vegetable. The property in certain plants, whence they are called sensitive, is obviously irritability, not sensibility. We cannot, indeed, conceive of sensibility where there is not intelligence. Sensibility is that property in the bodily organs by which we are apprized of the impressions made on those organs by external material substances. Yet this property always supposes a corresponding property in the mind, viz. the power of perception. It is in the correspondent exercise of these two powers that we become acquainted with the existence and characteristics of external things.

There is a certain kind of sensibility, which is common to all parts of the body to which nerves are supplied. This is varied in degree in different parts. There is also a specific sensibility in certain organs by which we recognise certain particular qualities in bodies. The facts on this subject are familiar, and the inferences perfectly obvious.

The sixth property among those enumerated is sympathy. The existence of certain relations between different parts, which relations were called sympathetic, has been already mentioned. Whether the name, sympathy, be well chosen or not, it is certain that parts placed at various distances from each other in living bodies do exercise mutual influences, besides those called nervous; and these are such as are not found to be exercised by different portions of inanimate matter on each other. The influences so exercised cannot be attributed to any of the properties of vitality already described. They must be attributed to a peculiar power, and to this so far as it has been noticed the term sympathy has been applied. Relations of this kind exist not only between different parts of the body, but also between the mind and the body. The full consideration of all that relates to this subject would extend this paper too far. It may perhaps be taken up at some future period.

Lastly, the intellectual powers, or the properties of mind are peculiar to animals, differing however very much in those of different species, and enjoyed in the greatest perfection by those of the human race. The consideration of these powers does not come within the ordinary limits of physiology. A few remarks will be made on them so far as they have an obvious influence on the body.

The connection of the physical and mental powers in the recognizance of external objects has been noticed. There is a similar connection exhibited in the performance of the voluntary motions. Mr. Stewart informs us, that by the power of volition we control our intellectual operations, calling up such thoughts, or trains of thoughts, as are requisite on any occasion for the purpose of comparison, or for any other purpose. Here the operation of that power takes place within the mind, and may not require the aid of any corporeal organs. But the same power of volition acts upon the body and is able to command the motions of certain parts of it. The motions are performed by the muscles, the mind furnishing only the stimulus, or exercising an influence analogous to that of a stimulus. Accordingly the mind has been said to exercise the stimulus of volition, when it produces action in the muscles. But the mind does not appear to act directly upon the muscles; for it does not act upon them, although it may exercise a volition, when the brain is deranged in certain modes. Nor, though the brain be altogether sound, can the mind act on the muscles, if the nervous connection between the brain and the muscles be destroyed. From this it appears that the nerves going to the voluntary or animal muscles have a power of transmitting the influence of the mind to the muscles, just as the nerves belonging to the organs of sense transmit to the mind the impressions made on those organs. This power, which certain nerves possess, of transmitting the commands of the mind, is one which has never received a name, and to which I shall not venture to give one. It is certainly something very distinct from the power of volition itself which resides in the mind.

The only remaining influence of the mind upon the body is that which it exercises by the power of sympathy. When the mind is engaged in the observation or consideration of subjects peculiarly interesting to it, the functions of the body are often affected, and that sometimes in a very powerful manner. Here we have the phenomena of passion. This subject has been very justly treated by Bichat, but he does not seem to have exhausted it. If the power of sympathy be made the subject of a future paper in this Journal, the display of it in the production of passion will be particularly noticed.—*New-England Journal.*

### CRITICAL ANALYSIS

